# Understanding How Pranic Healing Facilitators' Observed Experiences and Outcomes Arise: A Critical Realist Explanatory Model

**DOI:** 10.7759/cureus.112819

**Published:** 2026-07-16

**Authors:** Vinu V, Manasa B, Nagendra K Prasad, Siddharth Hiremath, Srikanth N Jois

**Affiliations:** 1 Research Center, World Pranic Healing Foundation, India, Mysuru, IND; 2 School of Social and Behavioral Sciences, Amrita Vishwa Vidyapeetham, Coimbatore, IND; 3 Department of Clinical Psychology, Manipal College of Health Professions, Manipal, IND

**Keywords:** holistic health, human biofield, lung health, traditional therapy, yoga and naturopathy

## Abstract

Pranic Healing (PH) is a naturopathic modality rooted in the biofield concept of *prana* (life force), employing non-touch techniques to normalize and balance the energy body to support well-being. While the primary focus of Pranic Healing facilitators (PHFs) is the improvement of recipient health, previous studies suggest that PH techniques of hand sensitization and pranic energization enhance practitioners' own well-being. This study aimed to explore PHFs' experiences during PH practices through qualitative exploration and a critical realist lens to identify the underlying generative mechanisms that explain how perceived energetic experiences are processed and contribute to healing outcomes. Twelve PHFs with 6-26 years of experience from six regions of India participated in in-depth telephonic interviews. Deductive and inductive thematic analysis, followed by retroductive reasoning to examine the empirical, actual, and real domains of experience, a layered explanatory model linking practitioner experiences, healing processes, and outcomes, were made. In addition to established PH-related experiences, two newly emergent categories among PHFs were recognized. PH functioned as a generative mechanism activating interconnected somatic, biofield, psychological, and consciousness-related healing processes. These findings provide an explanatory framework for understanding how PH practices generate perceived therapeutic and transformative outcomes leading to flourishing, which may further motivate practitioners to practice PH.

## Introduction

Energy medicine, closely tied to subtle energy, has been recognized in diverse cultural healing traditions. Referred to by diverse designations including *prana* (Sanskrit), *Qi* (Chinese medicine), and *Ki* (Japanese tradition), this fundamental life energy is conceptualized as flowing through every living creature, contributing to the maintenance of health and vitality [1,2]. According to Vedic knowledge (Atharva Veda, 3.31.9), *prana* supports a life of longevity, vibrant health, and spirituality [3]. The biofield, conceptualized as a dynamic energy and information field encompassing the body, is increasingly recognized in modern integrative and biomedical sciences for its role in regulating physiological and psychological functions [4]. Ayurveda describes *prana* as the vital life force that sustains and coordinates bodily, mental, and spiritual processes, maintaining health and well-being [2]. Together, these perspectives provide valuable insights into the mechanisms underlying healing, self-regulation, and the holistic process of health. Preclinical, clinical, and in vivo/in vitro studies on biofield and electromagnetic therapies suggest that ultraweak photon emissions may underlie biological regulation through coherent photonic and electromagnetic processes [5]. Biophoton emissions from the hands have also been shown to correlate with human intention and thought. The biofield therapy concept is rooted in the vitalist perspective, operating on the principle that healing occurs through biocommunication and/or energy transfer via the biofield. Biofield information acts as a bridge between mind and body, forming the foundation for understanding and facilitating mind-body interactions and healing [5,6].

The naturopathic biofield complementary method of Pranic Healing (PH) aims to treat physical and psychological conditions by balancing pranic energy in designated chakras or body zones, either via a Pranic Healing facilitator (PHF) or for personal healing [1]. The World Health Organization's Global Atlas of Traditional, Complementary, and Alternative Medicine (2005) recognizes PH as an energy-based healing modality [7]. Similarly, the Cochrane Library classifies PH under complementary, alternative, and integrative medicine [8]. Recent randomized controlled trials (RCTs) support its therapeutic potential, reporting benefits in depression reduction, improved sleep quality, relief of lower urinary tract symptoms, pain management, and enhanced healing of diabetic foot ulcers [9-12]. Beyond clinical settings, PH has also shown benefits in nonhuman domains. Laboratory studies report accelerated wound healing in HaCaT cell lines, while agricultural research demonstrates improved plant growth, yield, and gene-level changes [13,14]. These multidimensional benefits align with several United Nations Sustainable Development Goals (SDGs) [14].

Within the framework of Pranic Healing, developed by Master Choa Kok Sui, there are 14 fundamental principles. Empirical observations examined these principles across subjective, physiological, and experimental domains of principles including life force, pervasiveness, transmittability, contamination, controllability, cleansing and energizing, correspondence, interconnectedness, and directability [1]. Collectively, these PH principles describe *prana* as a dynamic, pervasive, and responsive subtle energy that can be absorbed from the environment, transmitted intentionally, influenced by mental states, and directed toward specific healing outcomes. Within PH theory, healing occurs through processes such as energetic cleansing, the replenishment of depleted energy, the modulation of chakras, and the intentional projection of *prana*. The core principles of PH, along with their attributes, functional roles, and underlying energetic processes, are presented with supporting empirical findings to elucidate the nature and influence of the biofield (*prana*) [1]. This integrated framework demonstrates the coherence between Pranic Healing principles, healing practice, and observed outcomes [9,10,13,15-21].

Empirical evidence spans qualitative, experimental, clinical, and review studies, providing support for several core principles and functions of Pranic Healing. Adolescents perceived air and ground *prana* differently, and positive perceptions were associated with greater well-being, supporting the concepts of life force, whereby *prana* nourishes, maintains, and facilitates healing, and pervasiveness, which proposes that *prana* is universally available and can be accessed through interaction with the environment [15,16]. Experimental studies have reported reproducible sensory experiences and physiological changes during pranic techniques, consistent with transmittability, the principle that *prana* can be intentionally transmitted, although the underlying mechanisms remain unclear [17]. Cell culture experiments demonstrating accelerated wound healing following the projection of multiple-colored *prana* illustrate controllability, whereby practitioners intentionally regulate pranic flow through focused intention, visualization, and pranic breathing [13]. Clinical studies linking chakra cleansing and energizing protocols with normalizing sleep, reduced depressive symptoms, and physiological correlates of chakra energy support the complementary functions of cleansing, which removes diseased or congested energy from the aura and chakras, and energizing, which replenishes fresh *prana* to restore energetic balance. These findings also align with the principle of contamination, which proposes that energetic congestion contributes to emotional distress, fatigue, and ill health [9,10,18]. A systematic review of biofield therapies reporting improvements in physical and psychological outcomes across diverse populations is consistent with correspondence, which posits that changes in the energy body are reflected in physical and psychological states and vice versa [19]. Similarly, a fibromyalgia case study demonstrating reduced pain and improved sleep following distant healing illustrates interconnectedness, the proposition that individuals remain energetically connected, permitting remote therapeutic influence [20]. Finally, experimental perception studies showing that participants could intentionally create, localize, and trace *prana* support directability, whereby *prana* can be precisely directed toward specific body regions or therapeutic targets through focused visualization and compassionate intent [21].

Pranic Healing sessions are conducted in a calm, structured, and distraction-free setting to facilitate participant comfort and receptivity. Participants are seated comfortably and guided to remain relaxed and attentive and open to their subjective experiences throughout the session. The healing process is delivered as a non-touch, non-pharmacological complementary practice and does not involve massage, physical manipulation, the use of needles, equipment, or psychological counselling. Depending on the needs of the participant, healing is conducted either in person or through distant healing procedures. The Pranic Healing facilitators (PHFs) are trained in applying structured PH protocols intended to support well-being and energetic balance. Their training progresses through basic, elementary, intermediate, and advanced levels, covering concepts related to *prana* and chakras, cleansing and energizing techniques, meditation practices, distant healing methods, and ethical discipline. Certification is obtained through supervised healing practice and documented healing experience. Participants are encouraged to maintain openness toward internal sensations and subtle experiential changes while minimizing distractions and unnecessary movements during the sessions (Appendices) [1].

Touching the tongue to the palate, deep breathing, setting intentions, and the focused awareness of sensations are common practices across many disciplines [6,22-24]. Prior to administering healing or utilizing its techniques, PHF typically performs an invocation to solicit divine blessings for healing and protection, underscoring its integral role [1]. Both PHF and recipients often report perceivable sensations of the biofield (*prana*) during the healing process, indicating an experiential awareness of energetic shifts [25]. Sensitizing the hands allowed research participants to experience different sensations [25,26]. Such subjective perceptions can be systematically evaluated in terms of both quantity and quality, particularly through the process of scanning [26]. In scanning, the PHF sensitizes their hands and sets a clear intention to assess the participant's energy field. Practitioners move their hands through the space surrounding the subject's body without making physical contact and interpret the sensations they perceive. By holding their hands where pranic energy sensations remain intense, they perceive the chakra and aura condition [10]. Imbalanced pranic energy levels manifest as protruding sensations known as pranic congestion or hollow sensations referred to as pranic depletion. Cleansing involves removing used-up energy from the affected area by sweeping the hands with the intention of disintegrating it and releasing it into salt water. Energizing, on the other hand, refers to projecting fresh *prana* toward the target area with healing intent [17]. This process involves consciously receiving pranic energy through the hands and chakras and then projecting it using the hands or fingers. Positive intention, combined with the visualization of specific colors as suggested in the PH manual, further enhances the healing process [13]. This integrated framework demonstrates the coherence between pranic theory, healing practice, and observed outcomes.

The PHF's mindful state plays a vital role, while hand sensitization, energization, and structured breathing further support the practice [21]. Psychosocial and spiritual qualities such as compassion, kindness, mindfulness, self-awareness, and reflective presence in the PHF help establish an optimal healing environment. Within this context, healing intention is understood as the mindful resolve of one or more individuals expressed through both intuitive and conscious action to promote the health of another person or oneself [27,28]. Mindfulness is characterized by present-moment, nonjudgmental awareness and may enhance interoceptive awareness, thereby facilitating greater perception of subtle pranic energy sensations [16]. In PH, intentional focus combined with compassionate awareness directs the practitioner's energy work, supporting emotional regulation and fostering empathetic connections with clients [1].

Despite the growing body of evidence, research on PH offers only limited insight into PHF lived experiences and the effects of sustained engagement in the practice. Using a critical realist lens and a process-oriented conceptual framework, this qualitative study aimed to develop a comprehensive understanding of Pranic Healing (PH) practice by analyzing facilitators' qualitative accounts of the healing process and its perceived outcomes.

## Materials and methods

Study design

This is a qualitative exploratory study employing thematic analysis within a critical realist framework, followed by the development of a conceptual model.

Sample

The study employed purposive sampling with 12 certified PHFs from six geographic zones of India, all of whom had formal training and practical experience and voluntarily participated in the study.

Inclusion and exclusion criteria

PHF includes being an Indian citizen over 18, having completed 48 hours of training for Associate Certified Pranic Healer (ACPH) (PH training and certification levels were provided in the Appendices), fulfilling the prerequisite of 25 healing cases for ACPH, and possessing a minimum of two years of experience. Practitioners who did not provide consent were excluded from participating in the study.

Procedure

Permission to conduct this exploratory study was first obtained from the Institutional Ethics Committee of World Pranic Healing Foundation (WPHF), India (approval number: 3/2022/16/7/2022). PHFs were recruited through purposive sampling by sending invitations via email to PH foundations across different parts of India, ensuring fair representation across six country zones. The World Pranic Healing Foundation in India served as the venue for the telephone interviews. We included facilitators from each zone who confirmed their participation. PHFs were assessed for eligibility by verifying their ACPH certificates. Participation was voluntary, with no compulsion of any sort, and confidentiality was maintained; no compensation was provided. PHFs underwent in-depth telephonic interviews in English with the three-member research team (one psychologist, one experienced pranic healer, and one researcher), and the interview duration ranged from 60 to 90 minutes. Their experiential, psychological, and self-developmental outcomes and practice-related dimensions of PHF's perspective were enquired using open-ended questions. Data were organized and analyzed using Microsoft Word 2016 and Microsoft Excel 2016 (Microsoft Corp., Redmond, WA). Details of the researcher are provided in the consolidated criteria for reporting qualitative research (COREQ) table (Appendices).

Tools

Sociodemographic Questionnaire

A sociodemographic questionnaire was used to collect the basic information of each PHF, such as name, gender, age, location, healing experience in years, and education and training, using Google Docs (Google, Inc., Mountain View, CA).

Open-Ended Questionnaire

This questionnaire was developed by the research staff of WPHF, India - Research Center (I-RC) and verified by a research expert. There were 13 open-ended questions asked to PHF through telephonic interviews (Appendices).

Data Analysis Procedure

To capture this layered reality, we adopted a multistage analytic process: (1) thematic analysis: inductive, theory-informed comparative thematic analysis [29,30]; (2) critical realist interpretation: a stratified analysis based on Roy Bhaskar's ontology of experience, events, and reality [31]; and (3) conceptual interpretation/model development: synthesis of findings into a process model or explanatory framework.

Initially, the audio recordings of the telephonic interviews were reviewed, and the transcripts were read repeatedly to ensure transcription accuracy and facilitate deep familiarization with the data. Open codes were inductively generated using in vivo coding with minimal preconceptions, focusing on participants' descriptions of energy sensations, healing interactions, awareness, emotional changes, behavioral experiences, and perceived outcomes. Conceptually similar codes were grouped into preliminary categories, and coding disagreements were resolved through iterative discussions until consensus was reached among all authors. The categories were subsequently compared with concepts reported in existing Pranic Healing literature while preserving novel experiential themes as distinct categories. Following the development of the final themes, the first and last authors undertook a theory-building phase guided by critical realism to explain the interrelationships among experiences, actions, and outcomes rather than generate additional themes. The emerging explanatory model was continuously refined through movement between the original transcripts, themes, and established Pranic Healing principles and relevant psychological theories, ensuring that all interpretations remained grounded in participants' accounts while representing theoretical inferences rather than empirically validated causal mechanisms.

Following Corbin and Strauss, open codes were generated inductively from participants' narratives and grouped into categories based on conceptual similarities, which were subsequently refined into broader themes and subthemes through iterative discussions and consensus among the authors [32]. The resulting thematic structure is detailed in the Appendices and supported by representative participant quotations. The emergent themes were then interpreted deductively in relation to existing Pranic Healing (PH) literature and concepts, followed by a retroductive critical realist analysis to identify the empirical (experienced phenomena), actual (observed outcomes), and real (underlying mechanisms) domains. Finally, these insights were integrated to develop an explanatory process model of the Pranic Healing facilitation process.

## Results

Sociodemographic characteristics

Table 1 illustrates the sociodemographic characteristics of 12 PHFs from various zones in India, with a mean age of 49±12.96 years and a mean experience of PHFs of 6-26 years. It found an equal distribution of male and female respondents, with the majority being married and all identifying as Hindu. Educationally, most PHF held postgraduate qualifications (n=9), including PhDs (n=2). A few PHFs were graduates (n=3), which included Bachelor of Medicine, Bachelor of Surgery (MBBS) doctors (n=2) and a Bachelor of Physiotherapy graduate (n=1). Additionally, PHF included specialists in allopathy and Ayurveda, medical doctors, and PH trainers (n=5). Geographically, all PHF resided in urban areas, with representation across different country zones. Professionally, 75% practiced PH full time, and 25% practiced part time.

**Table 1:** Sociodemographic characteristics of PHFs PHFs: Pranic Healing facilitators

Domain	Specifications	N (%)
Gender	Female	6 (50)
	Male	6 (50)
Marital status	Married	11 (91.6)
	Unmarried	1 (8.3)
Religion	Hindu	12 (100)
Education	Graduate	3 (25)
	Postgraduate	9 (75)
Locality	Urban	12 (100)
Country zones	South	2 (16.6)
	North	2 (16.6)
	East	2 (16.6)
	North Central	3 (25)
	West	2 (16.6)
	North East	1 (8.3)
Nature of practice	Full time	9 (75)
	Part time	3 (25)
Healers' experience in years	6-12	5 (41.6)
	13-26	7 (58.3)

Thematic analysis

**Figure 1:**
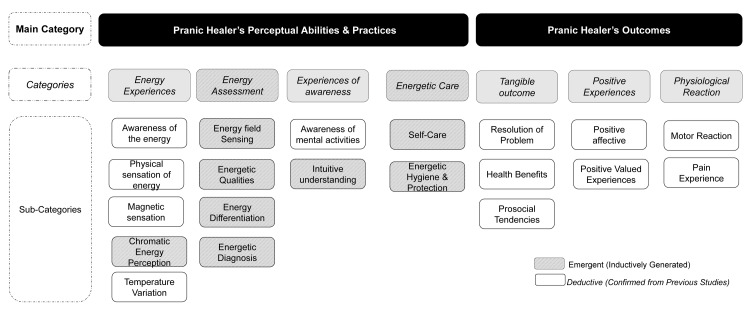
Thematic analysis of experiences of PHF This image is an original author-created schematic using PowerPoint (Microsoft Corp., Redmond, WA) and was not generated using AI PHF: Pranic Healing facilitator

In thematic analysis, codes were collaboratively grouped into subcategories, categories, and themes; it has been reviewed for coherence and refined for clarity and then defined and named to reflect their essence. This thematic analysis provided an empirical foundation grounded in participants' lived experiences (Figure 1).

Two main categories emerged from this process: pranic healer's perceptual abilities and practices and pranic healer's outcomes. The categories under pranic healer's perceptual abilities and practices were energy experiences, energy assessment, experiences of awareness, and energetic care. The categories under pranic healer's outcomes were tangible outcome, positive experiences, and physiological reaction (Figure 1). Subcategories, definitions, and codes were mentioned in the Appendices. Previous PH technique studies on Sensitizing the Hands Practice (SHP) and Meditation on Twin Hearts among students showed similar subdivisions [25,29,30]. This first qualitative study on PH facilitators introduced additional categories within healing experiences and practices, specifically energy assessment and energetic care. Further subcategories were identified: chromatic energy perception under energy experiences; energy field sensing, energetic qualities, energy differentiation, and energy diagnosis under energy assessment; and intuitive understanding under experiences of awareness. Subcategories of energetic care included self-care, energetic hygiene, and protection.

Critical realist application

**Figure 2:**
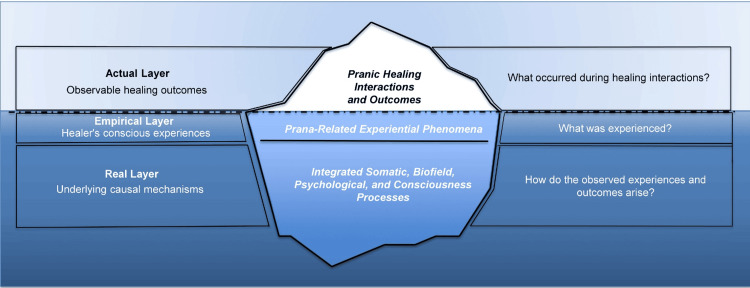
Critical realist stratified ontology of Pranic Healing facilitator This image is an original author-created schematic using PowerPoint (Microsoft Corp., Redmond, WA) and was not generated using AI

In the second stage, we applied critical realism's stratified model to deepen our understanding of each theme. The empirical layer captured participants' reported experiences; the actual layer examined what occurred during healing interactions, which included recurrent perceptual, cognitive, and practice-based processes; and the real layer theorized the underlying generative mechanisms that might explain the observed patterns. The empirical layer (observed experiences and outcomes), the actual layer, and the real layer (underlying mechanism) were mentioned in Figure 2. The inferred mechanisms were not directly observed or empirically tested in the present study; rather, they represent theoretical explanations derived from participants' accounts and informed by established Pranic Healing principles and relevant theories.

Empirical layer

Within the empirical domain, the figure captures PHF's conscious and subconscious experiences, including energy experiences, experiences of awareness, and perceived energy assessments. These experiences represent the phenomenological and interpretive dimensions of PH, wherein PHFs describe subtle energetic phenomena as directly perceived, embodied, and meaningfully differentiated through healing practice. The framework positions these accounts as experiential observations situated within the empirical layer of reality, reflecting how PHFs perceive and interpret their encounters during practice.

Actual layer

The domain covers PH-related events and their outcomes, regardless of observability or measurability. These include the resolution of problems, prosocial tendencies, health benefits, positive experiences, and positively valued experiences reported by participants. Within a critical realist perspective, such outcomes are understood as emergent tendencies arising within open and context-dependent systems through the interaction of PHF practices. Accordingly, the framework does not assume simple or deterministic causal relationships; rather, it recognizes that similar practices may generate variable outcomes depending on the conditions under which they operate. At the same time, the recurrence of patterned experiential and outcome-related regularities across participants allows for cautious explanatory inference regarding the operation of underlying generative mechanisms.

Real layer

The real domain advances explanatory inference by proposing deeper underlying mechanisms and causal powers that may underlie the PHF's experiences and outcomes identified across the empirical and actual domains. Through the synergy of PH principles, energetic hygiene, and Arhatic Yoga (AY), facilitators can attain energetic balance, self-actualization, and healing. These practices may interact dynamically, exerting a mutual influence that strengthens one another over time. Conditional factors, such as intrapersonal conditional factors and interactional mechanisms, enhance the therapeutic capabilities and outcomes within the practical realm for PHF. Within the framework, Arhatic Yoga may cultivate the PHF's inner qualities and energetic sensitivity, energetic hygiene may support the regulation and protection of the healing process, and PH techniques provide the operational healing methods [1]. Together, these mechanisms are theorized to generate the experiences and outcomes observed in the empirical and actual domains of PHF.

## Discussion

Empirical layer

Interpreting Biofield-Related Experiential Phenomena During Pranic Healing

Diverse sensory experiences within biofield practices are consistently observed among both recipients and PHF; notably, individuals without formal healing training who participate in guided biofield sessions have also described similar experiential occurrences [25]. PH builds upon the postulate that *prana* nourishes, maintains, and heals the physical and energy bodies, while its second principle proposes that *prana* is universally accessible through the environment and can interact with human energy fields and bodily systems [1]. Similar postulates were also reported in the complementary therapy of Tai Chi [33]. In the present study, PHFs reported experiential phenomena including energy awareness, physical and magnetic sensations, chromatic energy perceptions, and perceived temperature variations during healing practice. Importantly, these experiences are interpreted as experiential observations situated within the empirical domain of reality rather than as definitive evidence of underlying causal mechanisms. Similarly, the PH technique of practicing Sensitizing the Hands Practice (SHP) helps individuals perceive different pranic energy sensations, including magnetic, physical, thermal, tingling, pressure-like, and other subtle experiences. This process involves heightened awareness, focused attention, receptivity, and openness to experience, enabling PHF to become more sensitive to perceived changes in the biofield and energetic environment [16,17].

A newly emerging category within the empirical domain of this study was energy assessment experiences reported by PHF. These experiences included perceived areas of energetic depletion or congestion; localized sensations such as pricking, heaviness, pressure, or pain; and intuitive associations between perceived energetic disturbances and specific bodily regions or chakras [10]. Some PHFs described differentiated energetic qualities that they interpreted as corresponding to particular health conditions or dysfunctions within the recipient. Facilitators reported, "I can make it out the type of energy that I'm touching. So, that texture is different in many subjects." "I feel fresh energy and disease energy, and both have different feelings." In certain instances, PHFs reported experiencing transient sensations or discomfort in bodily areas corresponding to the client's affected organ or condition during the healing interaction. Such accounts reflect embodied and interpretive experiential observations situated within healing practice and are consistent with descriptions found in biofield and energy-healing literature concerning empathic resonance, energetic perception, and practitioner-reported assessment phenomena [34-36].

In the present study, PHFs reported experiential phenomena including energy awareness, physical and magnetic sensations, chromatic energy perceptions, and perceived temperature variations during healing practice. These experiential reports may reflect theoretical concepts associated with an emergent living-system-generated bioelectromagnetic field capable of transferring energy and information between biological systems [34]. Within this conceptual framework, such interactions have been proposed to contribute to phenomena such as empathic resonance, in which one individual emotionally, psychologically, or physiologically resonates with the state of another person [35]. These experiences may also relate to embodied perception, where bodily sensations and interoceptive awareness contribute to perception and meaning-making processes. Nelson and Schwartz (2005) suggested that individual differences in biofield awareness are associated with self-awareness, interpersonal sensitivity, conscious intention, and local bioelectromagnetic interactions [36]. Furthermore, subjective energetic sensations reported by PHFs may involve access-level consciousness, wherein bodily sensations become cognitively accessible during chakra scanning and are consciously interpreted as indicators of chakra size, diameter, or the intensity of perceived sensations [37]. Collectively, these perspectives provide a theoretical basis for understanding biofield-related phenomenology during PH. This layer therefore contributes to the postulation of the biofield concept and the PHF's perceived sensitivity in identifying subtle energetic changes during the healing process. This heightened sensitivity may enable facilitators to enter deeper levels of the healing process, where other PH principles may begin to manifest and interact as part of the deeper healing experience.

Actual layer

Observable Healing Interactions and Outcomes During the Pranic Healing Process

Pranic Healing protocols are intended to guide the PHF in the prioritization of energy balancing in terms of the application of techniques on chakras or energy bodies [1]. Deep-level PH processes may involve the expression of multiple postulated functions of *prana* (biofield), shaped by the nature of the client's condition and the PHF's expertise in addressing specific concerns. These dynamic healing interactions are perceived to contribute to beneficial outcomes, including transformative experiences within the PHF and positive changes related to the recipient's condition and overall well-being.

What Occurred During Healing Interactions

This research identified the consistent implementation of the PH process throughout different stages, adhering to the procedures outlined in the PH literature among PHF [1].

Energy Assessment Process

As a first step in the healing process, the PHF cultivated the awareness of *prana* by sensitizing and perceiving energetic sensations between the hands. This was followed by the energetic scanning of the chakras or affected bodily regions to identify perceived areas of congestion, depletion, or imbalance [1]. In patients with urinary tract symptoms, the scanning-based assessment of chakra energy levels showed significant imbalances before healing, whereas post-healing assessments indicated greater balancing across major chakras [10]. Comparable approaches for sensing and assessing the energy field have also been reported using AcuGraph, Pranic Scanning, and the Biofield Assessment Form [26,38,39]. Experiences of PH facilitators related to the scanning were "energetic scanning of chakras or affected regions, identification of perceived energetic congestion or depletion and blockage," etc. These accounts support the view that PHF subjectively perceives energetic imbalances during chakra scanning, functioning as an energy-based identification stage analogous to diagnostic assessment within the healing process. It is important to note that PHFs do not provide medical diagnoses; instead, they assess and interpret perceived energetic conditions within the biofield framework of Pranic Healing [1].

Normalizing the Energy Field and Chakras

Based on the PH principle, *prana* nourishes, maintains, and heals the body by rebalancing the energy body. Subsequently, the PHF performed cleansing procedures intended to remove dense or diseased energy, followed by energizing techniques aimed at projecting fresh *prana* into the affected regions. The cleansing of dense or diseased energy and energizing with fresh *prana* help to balance the pranic energy. Similar postulates were used by traditional Chinese medicine; acupuncture is based on the concept of balance and unobstructed flow of *Qi*; its blockage can lead to imbalance and disease [40]. In acupuncture, needles are inserted to restore the flow of *Qi* through the meridians and promote energetic balance within the body [41], whereas in PH, similar balancing processes occur through non-touch cleansing and energizing *prana* to the chakra or organ part with the help of intention and structured breathing [9,13]. Breathing in air introduces *Qi* from the outside into the lungs, resulting in lung *Qi* [41]. PHF regulates respiration akin to *pranayama* when receiving and projecting *prana* [17]. The above findings indicate that breath regulation may play a role in directing, enhancing, or projecting vital energy during healing processes. PH facilitators reported the following: "Scan and feel the density of energy, describes resistance and heaviness while cleansing congested chakras, and smoother movement as energy clears" (VKJ, RM, VKJ, and ARB); "Chakra clearing linked with smoother healing and energy flow" (SAL); "I can feel like the flow of energy has stopped, or I feel a pushback, which is quite common" (STL and ARB).

Facilitator-Recipient Energy Interaction

The facilitators reported experiences such as "pain in the kidney while healing a kidney patient," "pain in the arm during healing," and "pain sensations in the leg during healing." Facilitators also noted that "clients experienced 75% pain relief after sweeping and complete healing later" in conditions such as frozen shoulder and tennis elbow and that "during the pandemic, most healings were performed distantly." Within PH frameworks, such experiences are interpreted as manifestations of heightened sensitivity to the recipient's energetic or bodily disturbances during focused therapeutic engagement. The possibility of bioelectromagnetic interactions between individuals has also been proposed in healing contexts, including distant healing practices where facilitators attempt to direct healing intention toward recipients remotely [20].

Compassionate Intent of the Facilitator

PHF reported, "To relieve the pain and suffering of others, I started healing, found my life purpose as a healer. When we heal more people, I feel stronger, happier, and get satisfaction" (ANA). Compassion may further play an important role within facilitator-recipient interactions. Compassion received from others has been positively associated with self-compassion, resilience, reduced psychological distress, and greater flourishing and meaning in life [42]. Compassion, unconditional love, and caring intention are consistently described as central foundations enabling authentic healing and therapeutic connection [43]. In healing contexts, the PHF's compassionate intention while projecting energy toward chakras or affected regions may contribute to a greater sense of safety, care, and therapeutic support for the recipient. The founder of Pranic Healing advises touching the heart chakra during energizing to amplify projected energy, integrating *prana* with compassion and love [1]. PHFs are encouraged to cultivate compassion and self-awareness through the cultivation of virtues, particularly loving-kindness and noninjury, which form an integral component of Pranic Healing training, fostering compassionate intention and ethical conduct during healing interactions, such as Meditation on Twin Hearts, community service, and Arhatic Yoga, which strengthen the quality of healing intention and therapeutic engagement [44,45]. Healing is significantly influenced by compassionate resonance [35,36]. This indicates that compassion could be a key element in Pranic Healing by promoting a therapeutic bond, empathetic connection, and purposeful energy transfer.

The PH postulates relevant to the healing process (Table 2) include transmittability, contamination perceived by the PHF, controllability, cleansing and replenishment, correspondence, interconnectedness, and directability. These PH principles play a vital role and influence the PHF, as well as the recipient.

**Table 2:** Conceptual and empirical foundations of Pranic Healing (PH) techniques

Principles of Pranic Healing	Attributes	Function	Energetic Processes	Empirical Evidence
Life force	*Prana*, the vital force	Nourishes, maintains, and heals the physical and energy bodies	Absorbed from air, earth, sunlight, and food; circulated through chakras and meridians	Adolescents perceived air and ground *prana* differently and that viewing them positively influences their well-being [15]
Pervasiveness	Omnipresence of *prana*	*Prana* accessible everywhere; environmental interaction with energy fields and the body	Dynamic exchanges between the environment and the energy body, which interpenetrate and interact with physical matter	Individuals experience *prana* across contexts, reflecting its pervasive presence [16]
Transmittability	Transferability of *prana*	Enables healing at a distance or in-person through intention	Life force can be consciously projected or received	Pranic energy transfer is associated with distinct and reproducible experiential phenomena, and it can alter physiological state, and this leads to wellness [17]
Contamination	Energetic contamination	Results in energetic blockages, emotional distress, or fatigue	Balanced energy field supports resilience, health, and positive environmental interactions	Variations in chakra energy intensity are associated with physiological parameters in ultrasound and uroflowmetry reports (link between energetic imbalance and organ-level dysfunction) [10]
Controllability	Directed modulation of *prana*	Focused intention, visualization, and pranic breathing cycles	*Prana* responds to thought and will and in turn influences mental states	HaCaT wounds healed faster than controls when the healer projected colored *prana* [13]
Cleansing	Energetic cleansing	Cleansing removes dirty energy in chakras and aura	Removing and neutralizing stagnant energy to restore flow	Cleaning and balancing the solar plexus chakras induce sleepiness [9,10,18]
Energizing	Replenishment and revitalization	It replenishes fresh *prana* and restores energy in chakras and aura	Projection of fresh *prana* into specific chakras or regions	Energizing the basic chakra with pranic energy reduced the depression state [9]
Correspondence	Mind-energy-body connection	Energy body reflects the mind-body state and vice versa	Energy body and mind-body interact with *pranamaya* kosha via aura and chakras	Biofield applications across populations influence physical and psychological health [19]
Interconnectedness	Shared energetic field	Facilitates remote healing and mutual influence through shared etheric connections	Directed energy transmission through intention and adjustment based on perceived energetic response	Distant PH reduced fibromyalgia pain and stiffness and improved sleep after sessions [20]
Directability	Intent-mediated guidance of *prana*	Precise healing through focused visualization and compassionate intent	A thought-energy interface mediated by intention, attention, and mental imagery	Participants were able to create, place, and subsequently trace pranic energy, demonstrating its directed and sustained localization [21]

Reported outcomes across Pranic Healing applications

Resolution of Problems of Recipients

Under the theme of resolution of problems, facilitators described their experiences and observations of perceived improvements among recipients with psychological and physical conditions. PHFs reported working with individuals experiencing bipolar disorder and depression, which constituted most of their cases, and described an 85-year-old man with suicidal tendencies whose suicidal intent was perceived to have reduced after four healing sessions. Facilitators also reported observing perceived improvements in bladder stones within two to three months. During the COVID-19 pandemic, distant healing sessions were conducted, which recipients reportedly found valuable. Facilitators further described perceived benefits in cases involving arthritis, fear, insomnia, addiction, and cancer, among others (VKJ, SAL, STL, and KKJ). These accounts reflect facilitators' observations and were not independently verified through objective clinical outcome measures.

Important PH techniques include hand sensitization, the scanning of the energy field, the cleansing of contaminated or diseased energy, and energizing. Energizing involves projecting fresh *prana*, either as white *prana* or specific healing colors, depending on the nature of the ailment and the expertise of the PHF [46]. Green *prana* is used for cleansing congested energies, while blue is associated with calming, soothing, and reducing discomfort or emotional stress. Light whitish red is applied to enhance vitality and strengthen weak or depleted areas, whereas light whitish violet and electric violet are considered transformative and restorative. Multiple colors may be sequentially applied for wound healing, tissue repair, and functional recovery [46]. In mild and moderate depression, the basic chakra was activated to improve vitality, while the solar plexus chakra was inhibited using light whitish blue *prana* to reduce stress and promote relaxation. Similar applications were also extended to plant vitality and crop productivity. The specific use of PH color techniques and outcomes are provided in Table 3. Collectively, these color-specific interventions reflect the therapeutic intentions of PHF and the expected energetic changes associated with different healing objectives [9,10,13,17,18,20,47-49].

**Table 3:** Intention-specific application of Pranic Healing (PH) colors and associated outcomes PHF: Pranic Healing facilitator

Specific PH Colors During Energizing	Intention of PHF	Empirical Evidence
Light whitish red	To enhance vitality, normalize chakra activity, and restore energetic functioning	Mild and moderate depression reduced [9]
Light whitish red	To strengthen the plant and support growth	Enhancement in germination, root and shoot development, leaf structure, flowering, yield, and chlorophyll content [47]
Light whitish blue	To reduce emotional stress, induce calmness, and improve sleep	Improved sleep and stress reduction [9,10,18]
Light whitish blue	To soothe discomfort and reduce pain	Reduced bodily discomfort and pain in affected areas [10,20]
Multiple color	To accelerate tissue repair and optimize energetic support for wound healing	Multiple pranic colors are more effective for wounds or tissue repair compared to a single color [13]
Electric violet	To regenerate seeds/cocopeat/soil	Found 22% DNA polymorphism, along with root growth and increased yield in cabbage [48]
Electric violet	To transmute harmful energies and revitalize soil and plant systems	Enhanced shoot growth, leaf traits, flowering, and yield in banana [49]
No specific color	To send energy to the water bottle	Study participant felt energy sensations and reported energy moving out from hands and a mild forehead temperature reduction [17]

Health Benefits to PHF

The thematic analysis of health-related benefits revealed that PHFs reported perceived improvements following self-healing practices or healing received from fellow PHFs. These improvements were reported for conditions including severe wheat allergy, breathing difficulties, low hemoglobin levels, migraine, kidney stones, endometriosis, colitis, gluten sensitivity, and pharyngitis. PHF attributed recovery to self-directed practice or to the assistance of family and fellow PHF. These narratives are important because they reflect not only perceived benefits to recipients but also the PHF's own embodied experiences in the practice, which may strengthen commitment, confidence, and therapeutic engagement. The enhancement of subjective vitality through self-healing, along with mindfulness practices, has been found helpful in reducing symptoms associated with depression, anxiety, and anger-related conditions such as Hwabyung [50]. A guided loving-kindness meditation, followed by a guided breathwork exercise, changed biofield measures and indicated intra-subject correlations between different biofield measures [51]. Transcendental meditation may demonstrate potential viability for reducing anxiety and enhancing psychological well-being [52]. Autonomic self-regulation and transcendent states, assessed through EEG, psychological measures, and salivary biomarkers, are associated with enhanced immune function, reduced stress, and improved subjective well-being [53].

Transformative outcomes

Personal Growth and Healer Identity

Facilitators described Pranic Healing (PH) as a meaningful practice that fostered compassion, purpose, and personal growth. Many were motivated by a desire to relieve suffering and reported finding a sense of life purpose through healing. Personal healing experiences, spiritual connection with their teacher, and helping others contributed to greater acceptance, fulfilment, and meaning in life.

Moral Development Through Healing

Regular healing practice and service to others appeared to cultivate altruism, gratitude, humility, generosity, and social responsibility (KKJ, RJB, and ANA). Facilitators perceived that selfless service, charitable activities, and helping others not only supported healing but also enhanced their own compassion, satisfaction, and sense of purpose.

Trust and Receptivity in Healing Relationships

PHFs frequently demonstrated and guided energy-sensitivity practices (RM and VYL), enabling recipients to experience novel sensations and gain an experiential understanding of the subtle energy body. These experiences appeared to increase receptivity to the healing process and strengthen trust in both the facilitator and the practice.

Community Well-Being and Social Transformation

Consistent engagement in PH fostered compassion, embodied mindfulness, and social connectedness. Similar to findings reported in Yoga-based interventions, these qualities may contribute to enhanced well-being, prosocial attitudes, and community engagement [54]. Collectively, these processes may promote individual and community well-being through compassionate, trust-based, and service-oriented healing relationships.

Flourishing

Participants described experiences suggestive of psychological flourishing, characterized by personal transformation following engagement with PH, encompassing both hedonic well-being (feeling good) and eudaimonic well-being (functioning well and realizing one's potential). Hedonic well-being was reflected through experiences of happiness, peace, emotional relief, and improved emotional functioning, with participants reporting that they became "much happier," experienced "peace in my mind," felt "much better within myself," and noticed that "there is a 360-degree change in controlling emotions." Several participants described a reduction in emotional distress, noting that they no longer felt fearful, were less affected by stress, and could recover more quickly from adverse situations. Eudaimonic well-being was reflected through enhanced self-confidence ("I had the ability to shine like a star in all aspects"), greater self-understanding and acceptance ("there is lot of acceptance about people, about relationship, and about life"), a stronger sense of purpose ("found my life purpose as healer"), and increased competence in supporting others ("I correlate ailments with chakras to offer precise, effective healing support"). Participants also described feelings of empowerment ("being a pranic healer is very empowering"), meaningful contribution ("I can do some shift in a person's life through healing"), and improved psychological resilience ("it seems easier to detach from the entire situation and look at the whole scenario as a lesson or a journey").

The development of a PHF identity was another prominent aspect of flourishing, with participants describing profound personal transformation through healing practice ("my life has totally changed since I joined Pranic Healing"). Collectively, these experiences reflected not only enhanced positive emotional states but also personal growth, meaning, empowerment, and the realization of one's potential. Here, the highest level of well-being experiences by PHFs denoted flourishing due to the involvement of PH [55]. Flourishing among nursing students has been associated with the practice of energetic and mindfulness-based modalities, manifesting as enhanced emotional regulation, reduced anxiety, greater self-awareness, improved concentration and academic performance, increased resilience, physical well-being, productivity, mindful patient care, and a deeper appreciation of life and the surrounding environment [56]. Similarly, flourishing has been fostered through self-care practices such as healthy eating, adequate sleep, regular exercise, and mentor-supported well-being activities, which promote overall well-being [57]. In the present study, Pranic Healing appeared to operate through perceived processes of energy regulation and energetic balance, contributing to positive physical and psychological outcomes among recipients (Table 3) [49]. Drawing on these findings, it is plausible to propose that the activation of such underlying mechanisms may also contribute to emotional regulation, psychological resilience, vitality, self-awareness, and holistic well-being among Pranic Healing facilitators. Consequently, flourishing may be understood as an emergent outcome arising from the interaction of physical, energetic, psychological, and behavioral mechanisms within Pranic Healing practice.

Real layer

Generative Mechanisms Underlying PHF Experiences and Practice

**Figure 3:**
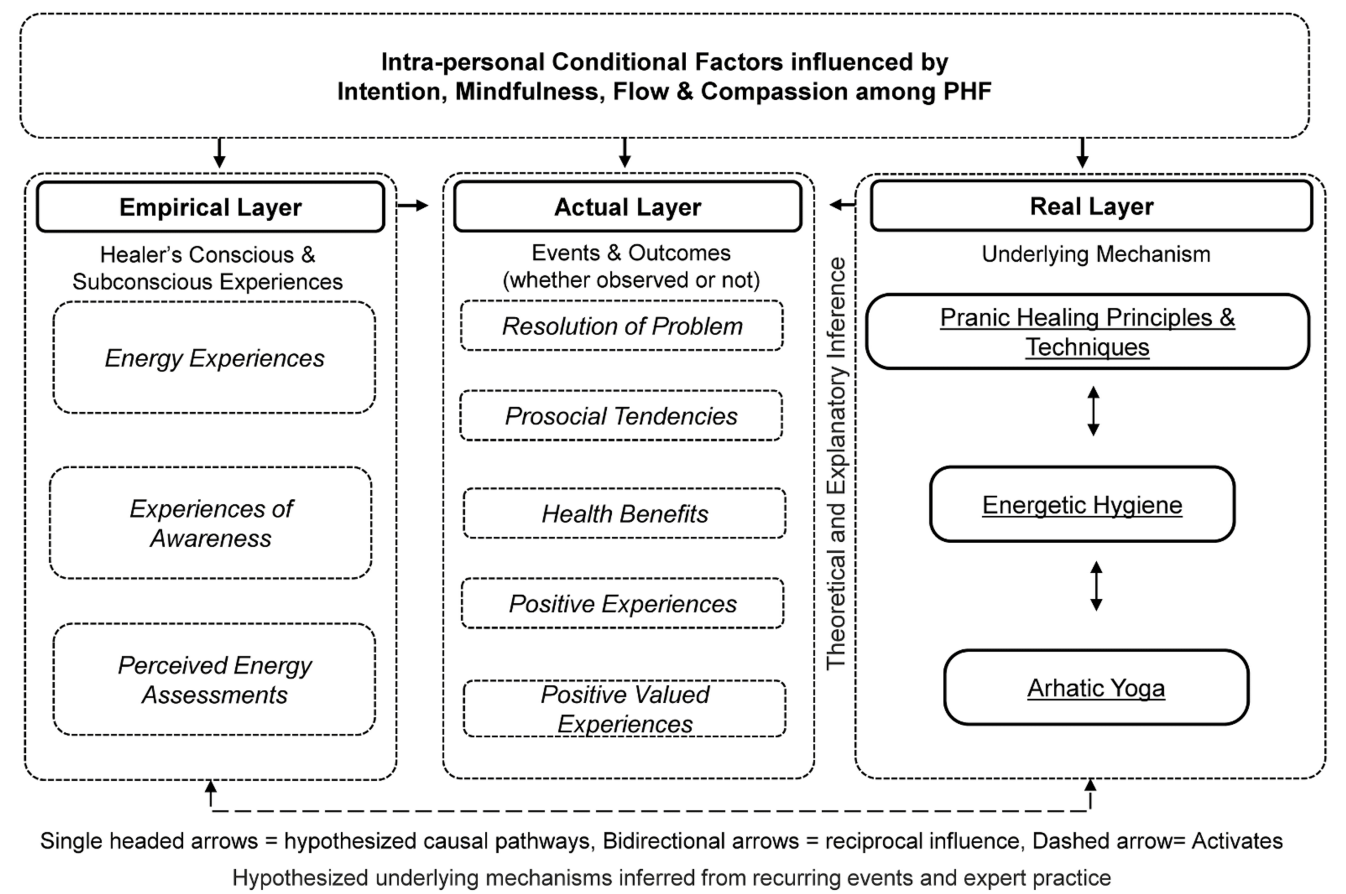
Inferred interactive generative mechanisms underlying Pranic Healing practice This image is an original author-created schematic using PowerPoint (Microsoft Corp., Redmond, WA) and was not generated using AI

Inferred interactive generative mechanisms underlying Pranic Healing practice: While applying PH techniques such as sensitizing the hands, scanning the aura and chakras of recipients or oneself, cleansing diseased energy, and applying energizing techniques, the fundamental principles of PH are believed to become activated [1]. Comparable concepts and theoretical models have also been described in other energy-based modalities, including auriculotherapy, acupuncture [33], *Qi*-based systems [24], and biofield-oriented healing approaches [58-61]. Within PH, these practices are associated with embodied perceptions of the biofield or *prana*. Positive intention, regulated breathing, focused attention, and the open awareness of pranic sensations may foster embodied mindfulness and a bidirectional mind-body connection [62]. In addition, empathetic-energetic resonance between the facilitator and recipient may contribute to changes in healing experiences, phenomenological awareness, biofield-related perceptions, and perceived healing outcomes [35,36]. Such experiences may facilitate an expansion of phenomenological consciousness and deeper experiential awareness (Figure 3). These conceptual hypotheses require further empirical investigation and validation before causal claims can be established.

The scanning process appears to involve cognitive functions such as attentional discrimination, sensory interpretation, and the evaluative awareness of pranic sensations while assessing perceived energy levels across different energy centers. This may reflect a form of access-level consciousness during the healing process [10]. Mindfulness and flow states are considered bidirectionally related, and deep embodiment characterized by heightened interoceptive and cross-sensory awareness [63] may support transitions from ordinary attentional states toward flow experiences [64] and higher-order conscious awareness [65]. Conditions such as meditation, breath regulation, mindfulness, contemplative awareness, intuition, focused attention, and related attentional practices may therefore contribute to heightened awareness and states of flow.

These processes may further promote psychological flourishing through enhanced self-awareness, emotional regulation, compassion, meaning, connectedness, and positive transformation, including strengthened confidence, hope, resilience, and positive emotional states among PHFs. Being receptive to love, kindness, and supportive interpersonal experiences while also cultivating self-compassion has been associated with psychological flourishing [66]. In the context of PH, such experiences may be facilitated through practices emphasizing mindfulness, compassionate awareness, loving-kindness, and self-regulation [64]. Expanded states of consciousness may also allow PHFs to report deeper intuitive understanding regarding recipients and healing processes [65].

At the same time, PHF-recipient energy interactions may be associated with physiological and experiential changes within the PHF [67]. Some PHFs reported unpleasant or adverse sensations during healing, including "skin irritation," "irritation while healing a kidney patient," "pain in the arm during healing," and "pain sensations in the leg during healing." Such experiences are often interpreted within PH as temporary energetic imbalances that can be managed through self-care and restorative practices. Commonly described methods include salt-water bathing, Meditation on Twin Hearts, physical exercises, energetic hygiene, and receiving healing from others [1]. PH frameworks also propose that PHF connect with divine or universal healing sources and facilitate the channelling of healing energy to support recipients' well-being [68].

Regular self-care and spiritual practices may additionally support sustained attention, mindful awareness, emotional regulation, clearer perception of pranic sensations, and deeper engagement in healing processes [69]. These practices may contribute to greater harmony between cognitive, emotional, physiological, and energetic dimensions of experience, thereby facilitating both personal growth and flourishing. In particular, Arhatic Yoga (AY) incorporates practices related to virtue development, breathing exercises, meditation, mindfulness, attentional discipline, personality development, compassionate community service, and the cultivation of higher awareness [1,29,44,45]. Emerging findings also suggest that AY practices may influence physiological domains, including the enhancement of beneficial microbiota associated with gut, immune, and gut-brain axis functioning [70]. Such multidimensional practices may strengthen flow states, enhance self-regulation, deepen conscious awareness, and contribute to psychological flourishing, potentially influencing both PH process and PH outcomes.

Preliminary technological approaches also provide indirect support for these concepts. AI-based aura visualization and emotion-detection models suggest that improvements in emotional and psychological states following sound healing may positively influence biofield regulation and energetic flow [71]. Similarly, perceived chakra energy levels assessed by facilitators were reported to become more balanced following PH interventions [10], while positive associations between biofield-related parameters and quality of life outcomes [21] further support a possible relationship between health status, biofield regulation, and pranic flow.

**Figure 4:**
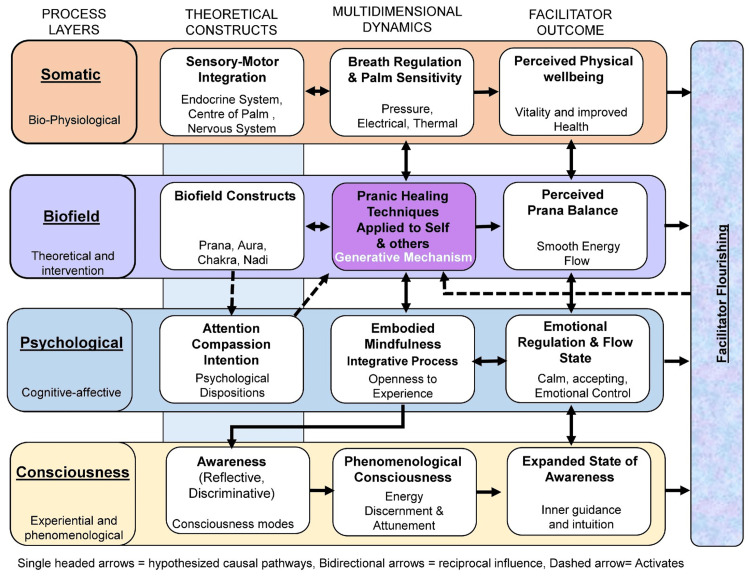
Proposed integrative multi-layered model of Pranic Healing facilitator This image is an original author-created schematic using PowerPoint (Microsoft Corp., Redmond, WA) and was not generated using AI

The thematic model (Figure 1) provided a detailed description of PHF's experiences, practices, and outcomes but remained largely descriptive. While it identified key themes such as energy experiences, energy assessment, awareness, and positive outcomes, it did not adequately explain the relationships among these domains or the mechanisms underlying them. The subsequent critical realist interpretation advanced the analysis by situating the findings within Bhaskar's empirical, actual, and real domains, highlighting the interaction between experiential, practical, and theoretical layers of reality [31]. However, this interpretation primarily emphasized the stratified nature of reality and generative mechanisms without explicitly integrating the psychological, psychobiological, and consciousness dimensions relevant to biofield-related practices [6,19,53]. The present model therefore reinterprets and extends the realist framework by integrating these additional layers, providing a more comprehensive account of the mechanisms and experiences associated with Pranic Healing facilitation (Figure 4).

Proposed Integrative Multi-layered Model of Pranic Healing Facilitator

It emphasizes the reciprocal interactions among somatic processes, biofield constructs, psychological dispositions, and consciousness-related experiences. Within this framework, Pranic Healing techniques are conceptualized as the central generative mechanism that activates healing-related processes, while embodied mindfulness functions as an integrative process that cultivates embodied awareness and energetic perception.

Pranic Healing Techniques as the Central Generative Mechanism

At the core of the model, Pranic Healing techniques applied to oneself and others function as the primary generative mechanism that activates healing-related processes across somatic, biofield, psychological, and consciousness domains. These practices are informed by biofield constructs such as *prana*, aura, chakras, and *nadis* while simultaneously engaging the psychological dispositions of attention, compassion, and intention. Through repeated engagement, Pranic Healing techniques facilitate the emergence of multidimensional experiences and outcomes that extend beyond the immediate healing context.

Embodied Mindfulness as an Integrative Process

Embodied mindfulness serves as the central integrative process linking healing practices with PHF's lived experiences. Characterized by openness to experience, present-moment awareness, and heightened sensitivity to internal and external phenomena, embodied mindfulness enables PHF to actively engage with bodily, emotional, energetic, and consciousness-related experiences. The reciprocal relationship between embodied mindfulness and Pranic Healing practice suggests that mindfulness enhances the quality of healing engagement, while repeated healing practice further strengthens embodied awareness and experiential sensitivity.

Somatic Processes and Sensory-Motor Integration

Within the somatic domain, healing practice is associated with sensory-motor integration, breath regulation, and enhanced palm sensitivity. PHFs frequently report sensations such as pressure, tingling, warmth, coolness, electrical sensations, and subtle movements of energy. The center of the palm functions as a primary interface for perceiving these experiences, integrating sensory input with nervous system activity and bodily awareness. Through repeated practice, this process may enhance sensory discrimination and contribute to perceptions of physical vitality, health, and well-being.

Biofield Constructs and Perceived Energy Regulation

Within the biofield domain, PHFs engage with theoretical constructs including *prana*, aura, chakras, and *nadis*, which provide a framework for interpreting healing experiences. These constructs guide healing practices and influence how PHF understands perceived changes in energy flow and energetic balance. The reciprocal relationship between healing experiences and biofield concepts suggests that experiential validation may reinforce the meaningfulness and application of these constructs in practice.

Mindfulness can be incorporated into virtually any activity, including the practice of Pranic Healing, as healers are required to direct sustained attention to their palms and observe subtle sensations perceived during the healing process. The nature and intensity of these sensations may vary across individuals and cannot be anticipated without such attentive awareness [72]. Such focused awareness may enhance the perception of faint tactile experiences, consistent with evidence that mindfulness meditation can increase sensitivity to subtle midair tactile perception [73]. Furthermore, mindfulness has been associated with multiple dimensions of embodied experience, including emotion processing, observational and non-observational body awareness, and both conscious and unconscious aspects of interoception [74]. Recent findings also indicate that sustained contemplative practices involving breathing may heighten sensitivity to embodied sensations by enhancing interoceptive awareness. This process may facilitate the integration of mind and body, enabling individuals to construct personal meaning from their bodily experiences. Such practices may also promote a shift from deliberate control to absorbed awareness, activate self-regulatory processes, and cultivate nonevaluative awareness, thereby supporting flow, cognitive reorganization, and reflective capacity [75]. Accordingly, mindfulness-related attentional and interoceptive processes may represent one plausible complementary explanation for the reported experiences and may operate alongside the proposed mechanisms of Pranic Healing rather than being mutually exclusive.

Psychological Processes: Attention, Compassion, Intention, and Emotional Regulation

The psychological domain encompasses the dispositions of attention, compassion, and intention, which shape the practitioner's orientation toward healing. These processes interact with embodied mindfulness to support emotional regulation, emotional balance, acceptance, and flow states. Through sustained practice, PHF may develop greater self-regulation, emotional stability, and psychological resilience, enhancing both personal well-being and healing engagement.

Consciousness Development and Expanded Awareness

Within the consciousness domain, healing practice supports the development of reflective and discriminative awareness, which contributes to phenomenological consciousness and the discernment of subtle experiential qualities. Over time, PHF may report expanded states of awareness characterized by heightened intuition, inner guidance, interconnectedness, and deeper insight into healing experiences. These consciousness-related processes help PHF interpret and integrate their experiences and may contribute to self-transcendence, personal transformation, and a broader sense of meaning and purpose.

Reciprocal Dynamics and Facilitator Flourishing

The model proposes reciprocal relationships among somatic, biofield, psychological, and consciousness-related domains, suggesting that these processes mutually influence and reinforce one another. Through sustained engagement in Pranic Healing practice, these multidimensional interactions may facilitate psychophysiological, biofield, and consciousness integration, thereby contributing to PHF development, expanded awareness, and facilitator flourishing, which may further reinforce engagement with Pranic Healing practice.

Strength

This study is strengthened by its inclusion of in-depth interviews with experienced, well-educated, and more full-time PHF. Their breadth of PH experience enabled the collection of rich qualitative data, supporting the development of theoretically informed propositions and potential mechanisms that contribute to the emerging biofield literature and inform future empirical research in PH.

Limitations

The proposed mechanisms were inferred from participants' subjective and retrospective accounts and were not directly measured or empirically tested. The study sample was relatively homogeneous, consisting of experienced Pranic Healing facilitators from India who shared similar cultural backgrounds and participated voluntarily. Consequently, the findings may not fully represent the perspectives of novice practitioners, individuals from different cultural contexts, or those engaged in other healing traditions, thereby limiting their transferability. Furthermore, participants' prior training, long-term practice, and commitment to Pranic Healing may have shaped their interpretations and experiences. Future research should include more diverse populations, comparative cultural settings, and mixed-methods approaches to examine the broader applicability and underlying mechanisms of these findings.

Implications

This study extends the discussion of complementary therapies beyond symptom management to include broader dimensions of human flourishing, self-development, and transformative experiences. The proposed framework should be viewed as a preliminary conceptual model that generates hypotheses for future research rather than definitive causal explanations. Future mixed-methods, longitudinal, and experimental studies are needed to examine the proposed mechanisms, pathways, and outcomes; integrate psychological and physiological measures; and further strengthen the evidence base for Pranic Healing and related biofield practices.

## Conclusions

The thematic analysis identified two main categories: pranic healer's perceptual abilities and practices and pranic healer's outcomes. Within this critical realist framework, PHF experiences were understood as emerging from interactions across interconnected somatic, biofield, psychological, and consciousness-related domains. The proposed multi-layered model further illustrates how facilitators perceive their growth and flourishing through engagement with Pranic Healing practices. From this critical realist perspective, Pranic Healing facilitation acts as a generative mechanism through which interactions across these domains may contribute to personal development, prosocial engagement, and human flourishing while providing a foundation for the future empirical investigation of the processes and mechanisms underlying these experiences.

## Figures and Tables

**Figure 5:**
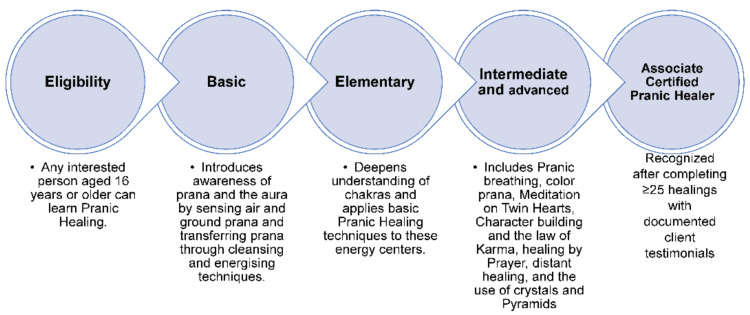
Supplementary figure: Pranic Healing training and certification levels This image is an original author-created schematic using PowerPoint (Microsoft Corp., Redmond, WA) and was not generated using AI

## Appendices

Table 4 shows the consolidated criteria for reporting qualitative research (COREQ).

**Table 4:** Supplementary file 1: consolidated criteria for reporting qualitative research (COREQ)

Number	COREQ Item	Guide Question	Your Response
Domain 1: research team and reflexivity			
1	Interviewer/facilitator	Which author(s) conducted the interviews?	2, 3, and 5
2	Credentials	What were the researchers' qualifications (e.g., PhD and MSc)?	MSc, PD and certified Pranic Healer, and PhD
3	Occupation	What was their occupation during the study?	Research and teaching
4	Gender	Male/female/other	4, M; 1, F
5	Experience and training	Previous qualitative research experience or training	Yes
6	Relationship established	Was a relationship established before data collection?	No
7	Participant's knowledge of the interviewer	What did participants know about the researchers?	Research investigators
8	Interviewer characteristics	Biases, assumptions, interests, and reflexivity	A professional interest in understanding participants' lived experiences
Domain 2: study design			
9	Methodological orientation	Grounded theory, phenomenology, thematic analysis, etc.	Thematic analysis along with critical realism (CR)
10	Sampling	Purposive, convenience, snowball, etc.	Purposive
11	Method of approach	Face to face, email, telephone, etc.	Telephonic
12	Sample size	Number of participants	12
13	Nonparticipation	Refusals/dropouts and reasons	Nil
14	Setting of data collection	Home, clinic, online, workplace, etc.	Workplace or home
15	Presence of nonparticipants	Was anyone else present during interviews?	Nil
16	Description of sample	Demographic and relevant participant characteristics	Yes
Data collection			
17	Interview guide	Were interview questions/pilot testing described?	Semi-structured interview guide
18	Repeat interviews	Were repeat interviews conducted?	No
19	Audio/visual recording	Audio/video recording used?	Audio
20	Field notes	Were field notes taken?	Yes
21	Duration	Length of interviews/focus groups	30-90 minutes
22	Data saturation	Was saturation discussed?	Yes
23	Transcripts returned	Were transcripts returned for participant checking?	No. Interview transcripts were not returned to participants for review or correction
Domain 3: analysis and findings			
24	Number of data coders	Number of researchers involved in coding	1, 2, 4, and 5
25	Description of the coding tree	Was the coding framework described?	Yes
26	Derivation of themes	Inductive, deductive, or hybrid?	Inductive and deductive
27	Software	NVivo, ATLAS.ti, MAXQDA, manual, etc.	Manual with Excel
28	Participant checking	Did participants comment on findings?	Experienced pranic healer in the team (investigator)
Reporting			
29	Quotations presented	Were participant quotations included and identified?	Yes
30	Data and findings consistent	Were the findings supported by the data?	Yes
31	Clarity of major themes	Were major themes clearly presented?	Yes
32	Clarity of minor themes	Were diverse or contradictory cases discussed?	Yes

Table 5 shows the open-ended questions asked to Pranic Healing facilitators during telephonic interviews.

**Table 5:** Supplementary file 2: open-ended questions

Open-ended questions
1. How have you felt as a pranic healer?
2. What drew you to frequently practice Pranic Healing?
3. In your experience, which health condition has benefited the most from Pranic
Healing sessions?
4. Did you have any experiences during Pranic Healing? If so, where in the body do
you feel them?
5. Describe your scanning experiences.
6. Do you use any tools to improve the efficacy of your Pranic Healing practice?
7. What methods have you found useful in increasing attention during Pranic Healing
techniques?
8. What are your strengths and weaknesses in practicing Pranic Healing?
9. Have you ever felt uncomfortable as a pranic healer? If so, how do you cope with
discomfort?
10. Have you noticed any differences in your life as a result of persistent Pranic Healing
practice?
11. How open are your clients and their families to receiving Pranic Healing?
12. Describe the experiences narrated by Pranic Healing subjects.
13. Could you tell me about your notable Pranic Healing case?

Figure 5 shows the Pranic Healing training and certification levels.

Table 6 shows the thematic structure and definitions.

**Table 6:** Supplementary file 3: thematic structure and definitions

Main Categories	Categories	Subcategories	Definitions	Codes	Healers/Participants
Category 1: healer's perceptual abilities and practices	Energy experiences	1. Awareness of the presence of energy	Pranic healer's conscious recognition of subtle energetic phenomena within or around their body	"I experienced the aura. I experienced the energy." "I was sensitive enough to feel the energies." "I feel the flow of energy for sure." "I can feel the energy. I can feel some energy passing through my hand…." "I can feel a lot of energy coming out of my hands freely." "I feel as much of my energy flowing out of my hand." RJB: "…I used to feel strange… I can feel energy flow. …feel the downpour of energy in my crown and the rest of the body…."	VKJ, STC, VPL, KKJ, RM, MGMNI, STL, and VPL
		2. Physical sensation of energy	Tangible, sensory dimension of energy interaction, as registered by pranic healers	"Tingling sensation, some current sort of sensation." "I felt something tingling on hand…." "Heaviness sensation." "I feel my crown becoming heavy." "There was a little air pressure I have to felt on my hand." "I feel pressure when I move my hand while cleansing." Hand movements are slow and heavy during cleansing. …heaviness in hand, electric current in my hands…." "…Tingling sensations in my crown area." "…Feel pressure when I receive energy before projecting."	SAL, VPL, ANA, RJB, VKJ, VYL, KKJ, and SAL
		3. Awareness of temperature variation	Pranic healer's conscious perception of changes in temperature as an aspect of sensing and working with pranic energy	"At times, temperature difference is there…." "If I'm doing healing for anger, usually it feels a little warm." "Warm or hot sensation in the solar plexus (anger) …," "…Heat or warmth…energy sensations…"	RM, ARB, and VYL
		4. Magnetic sensation	The perceived subtle forces such as magnetic attraction or repulsion felt between the hands while sensing or working with *prana*	Some magnetic thing like sensation during scanning	STC
		5. Chromatic energy perception	Perceiving and visualizing colors in the energy field during Pranic Healing	"Found diseased energy in grey color." "While projecting dark orange…projected a specific color *prana*…only pink *prana* felt…." …Project such different color *prana* in a darker mode…not there when I project whitish *prana*, …used to project that certain color *prana*…but feel pink."	ANA, RJB, VYL, and RJB
	Experiences of awareness	1. Awareness of mental activity	The conscious ability to notice and regulate one's own inner processes, such as thoughts, emotions, attention, and mental imagery	"I prefer to have silent and comfortable healing place (room), so no one will disturb during the healing." "I don't prefer hurry-burry healing. I used to spend some relaxed time before I take up any healing sessions. When I sit in a group, I easily become distracted. So, I prefer doing my healing back at my place. When I was passed through some stress condition…if healing sessions in the morning are not done, I feel irritated and anxious on that day. I playing a mantra, preferably Om, that helps the healer to concentrate and focus on the healing. During the healing, if we can focus on the patient's recovery, if the picture is very clearly in the healer's mind, this is the outcome which we want to achieve at the end of the healing	ANA, MGMNI, RJB VPL, and SAL
		2. Intuitive understanding	The healer's ability to spontaneously receive inner guidance, such as words, answers, or impressions, related to the client's condition or the healing process	Hearing inner guidance or words related to the client's condition and receiving inner answers, intuitions for doing healing	ARB
	Energy assessment	1. Energy field sensing	Pranic healer's initial step in scanning and sensing the aura, chakras, and areas of specific body parts using palms to detect the energy field	"Scan and feel the density of energy" describes resistance and heaviness while cleansing congested chakras and smoother movement as energy clears. "Warm or hot sensation in the solar plexus…."	VKJ, RM, VKJ, and ARB
		2. Energy differentiation	Distinguishing different types or conditions of pranic energy through sensory perception	"I can make it out the type of energy that I'm touching. So, that texture is different in many subjects." "I feel fresh energy and disease energy, and both have different feelings"	
		3. Energy qualities	Distinct characteristics or textures of pranic energy perceived by a healer	"Cactus-like sensation." "Energy texture is different in many patients. "Mentions detecting 'greasy substances' inside the chakra, implying spatial energy sensing, describes energy as 'dirty' that clears over time." "Greasy substances inside the chakra." ARB: "…Itchy energy in lungs, solar plexus, liver…." "I started scanning, and I feel some pinching sensation in my finger. And I can feel the different textures like a sandy texture or a feather texture during my scanning."	SAL, STC, VKJ, and SAL
		4. Energetic diagnosis	Interpreting sensory information to assess the client's energetic and health condition for targeted healing	Links emotional state (anger) to warmth. SAL/VKJ: distinguish congested chakras from cleared ones through sensation. Differentiates aggressive emotional energy by skin reaction. "…Can feel like the flow of energy has stopped…." "Stress feels pokey." "Felt some lumps…constipation." "Problem in kidney area…confirmed stone." "Marked lung issue…confirmed pulmonary edema." "Brain scan…felt warming." "Doctors suspected spleen…I found healthy chakras…later confirmed autoimmune." Chakra clearing linked with smoother healing and energy flow	RM, VPN, SAL/VKJ, RM, STL, MGMNI, RJB, STC, ANA, and SAL
		5. Motor reactions	The involuntary physical movements or muscular responses experienced by the healer	SAL: hand motion transitions from slow/heavy to smooth during healing. SAL: chakra clearing linked with smoother healing and energy flow	
		6. Visceral experiences		"Skin irritation." "…Irritation while healing a kidney patient"	VPN and ANA
		7. Pain	Subjective, unpleasant sensory experience of discomfort or ache that arises in the healer's own body during the healing process	Pain in the kidney while healing a kidney patient. Sometimes, I felt pain in the arm during healing. I have experienced pain sensations in my leg during healing	ANA, STL, and RJB
	Energetic care	1. Self-care	The deliberate and consistent actions taken by pranic healers to maintain their energetic well-being	"Before and after healing, I focus on cleaning my chakras, breathing techniques, and keeping the healing room energetically clean. I regularly take saltwater baths." "I sometimes do Twin Hearts Meditation or chant 'Om Shanti Om' while healing. When I am emotionally unstable, I avoid healing and pass the case to another healer, as I feel I cannot do justice to the patient at that time. I practice self-healing, take saltwater baths, soak my feet in salt water, and perform the Twin Hearts Meditation regularly. I also continue to reflect inwardly, as I believe inner reflection is a powerful tool, and I avoid unnecessary talking to maintain emotional balance"	SAL, VYL, and STC
		2. Energetic hygiene and protection	The PH techniques employed by healers to cleanse, protect, and strengthen their own energy field; purify healing tools; and maintain a safe healing environment	"I use crystals such as rose quartz, crystal disintegrators, and other tools to enhance healing, and I perform invocation and meditation before healing to connect deeply with the patient and energy system." "I perform daily invocation before healing, use cleansing after contaminated cases, and follow purification practices to maintain energetic clarity. I have not experienced contamination, possibly due to safety measures like meditation and cleaning with isopropyl alcohol"	VKJ, VPL, and RM
Category 2: healer's outcomes in Pranic Healing	Tangible outcome	1. Resolution of the problem	A client's noticeable relief from physical, psychological, or emotional issues and improvement in well-being	Client experienced 75% pain relief after sweeping and complete healing later (frozen shoulder and tennis elbow). During pandemic, most of the healings were done distantly. Treating bipolar disorder and depression, with most cases being psychological. Healed an 85-year-old man with suicidal tendencies, reducing his intent within about four healing sessions. I have also seen bladder stones reduce or completely disappear in two to three months, achieved good results in corona cases, and worked successfully with arthritis, fear, insomnia, addiction, and many cancer cases	VKJ, SAL, STL, and KKJ
		2. Health benefits	The healer's noticeable relief in physical health, psychological balance, and emotional stability and improvement in well-being.	"I was diagnosed with the severe wheat allergy; it was gluten allergy. Hemoglobin levels quite low and I had little bit problems and difficulty in breathing. I started with my own healings. I am come out from the wheat allergy. I got healed miraculously. So, there is no trace of endometriosis past 12 years now". "I was suffered at that time with a kidney stone. So, doctor suggested me for laparoscopic operation. My father and his fellow pranic healer worked on me one month for relieving the stone trough Pranic Healing. And finally, stones were not there." "In year 2003, I was detected colitis. Some other family members helped me doing Pranic Healing, and it was gone within 15 days." "Completely out of migraine. My medicines were stopped. And it took me around six months of time to heal pharyngitis"	RJB, STC, VKJ, and RM
		3. Prosocial tendencies	The tendency to help and care for others	To relieve the pain and suffering of others I started healing, found my life purpose as a healer. When we heal more people, I feel stronger, happier, and get satisfaction. Felt strong attachment to their Master, seeks guidance in dilemmas, and shows a deep desire to help patients through extended healing sessions. "I felt motivated to learn Pranic Healing after being healed myself of multiple health and personal issues over several years. I have a desire to help others following my father's suicide"	ANA, MGMNI, and STL
	Positive experiences	1. Positive affective experiences	The positive affect and pleasant emotions, feelings, and moods that healers report as a natural result of practicing Pranic Healing	Grateful, confident, and blessed. Stronger and happier after healing more people. "Feeling grateful and experiencing peace of mind." "Felt better after healing sessions and gained mental satisfaction and at times felt powerful and stronger as a healer." "Pranic Healing has made me more confident, practical in life, and given me new hope and motive. It has helped me believe in self-love and maintain better emotional control." "Being a pranic healer is very empowering. I feel free from limitations, both within and outside, and I have the ability to shine like a star in all aspects." "Seeing patients recover and knowing I have contributed to their improved health and happiness brings me contentment and joy"	ANA, MGMNI, STL, RJB, VYL, and SAL
		2. Positive valued experiences	The experiences of enhancing values, purpose, spiritually enriching insights, and life satisfaction as a result of persistent Pranic Healing practice	Before joining Pranic Healing, I was depressed and hostile, but now, I have better control over my emotions and good relationships with my family. Pranic Healing has made me calmer, more patient, more accepting of people, relationships, and life. I no longer cry when problems arise, and I handle challenges with greater emotional stability. I also recognize that glamor can be a weakness for healers, as knowing techniques can lead to a sense of superiority, and I consciously avoid falling into that trap	RJB and STC
	Physiological reaction	1. Motor reactions	The involuntary physical movements or muscular responses experienced by the healer	"I often feel the pushback from the patient chakra." "As the energizing is done, I can feel like the flow of energy has stopped, or I feel a pushback, which is quite common"	STL and ARB
		2. Pain experiences	Subjective, unpleasant pain experience felt to hands or body parts by the healer during healing	Pain in kidney while healing a kidney patient; sometimes, I felt pain in the arm during healing, I have experienced pain sensations in my leg during healing	ANA, STL, and RJB
